# Bilateral Earlobe Purpura as an Unusual Initial Manifestation of Systemic Lupus Erythematosus: A Case Report

**DOI:** 10.7759/cureus.118549

**Published:** 2026-10-25

**Authors:** Lucy Atherton

**Affiliations:** 1 Internal Medicine, Whittington Hospital, London, GBR

**Keywords:** autoimmune, hemophagocytic lymphohistiocytosis (hlh), mycophenolate therapy, rituximab therapy, sle, systemic lupus erythematosus, thrombocytopenia

## Abstract

Systemic lupus erythematosus (SLE) is a chronic, multisystem autoimmune disease characterized by autoantibody production, particularly antinuclear antibodies (ANAs). The broad clinical spectrum and absence of pathognomonic features can make diagnosis challenging, particularly when alternative autoimmune, infectious, and hematological conditions must be considered. We report a case of a 31-year-old woman who presented to the emergency department with a two-week history of bilateral earlobe purpura. Initial investigations demonstrated severe thrombocytopenia and normocytic anemia, and subsequent imaging revealed widespread lymphadenopathy. Early multidisciplinary assessment by acute medicine, hematology, and rheumatology, together with exclusion of hematological malignancy and positive autoimmune serology, supported a diagnosis of SLE. Despite initial treatment with intravenous immunoglobulin (IVIg) and corticosteroids, the bicytopenia persisted. Mycophenolate mofetil was commenced; however, worsening cytopenias and concern for secondary hemophagocytic lymphohistiocytosis led to the initiation of rituximab infusions. Her platelet count subsequently improved, and she was discharged with rheumatology and hematology follow-up. This case highlights an unusual cutaneous presentation of newly diagnosed SLE and the diagnostic challenges associated with severe hematological involvement at initial presentation. It also demonstrates the importance of multidisciplinary assessment and timely escalation of immunosuppressive therapy in severe disease.

## Introduction

Systemic lupus erythematosus (SLE) is a chronic multisystem autoimmune disease. Clinical manifestations are diverse and can involve the skin, musculoskeletal system, kidneys, nervous system, and hematological system. The 2019 EULAR/ACR classification criteria for SLE comprise a weighted scoring system incorporating the presence of ANAs, seven clinical domains (constitutional, hematological, neuropsychiatric, mucocutaneous, serosal, musculoskeletal, and renal), and three immunological domains (antiphospholipid antibodies, complement proteins, and SLE-specific antibodies), with individual criteria assigned weights ranging from 2 to 10 points [1]. Patients with a score of ≥10 are classified as having SLE. Because of the breadth of potential manifestations, diagnosis can be challenging, particularly when patients present without the classical clinical features of SLE [2]. Additionally, several autoimmune, infectious, and hematological conditions can mimic SLE. Diagnosis is generally based on clinical and serological findings, with autoantibodies such as ANA and anti-dsDNA most commonly detected in patients with SLE using immunofluorescence assays or enzyme-linked immunosorbent assays [3]. Anti-Sm autoantibodies are less common but highly specific for SLE and are one of the immunological diagnostic criteria for the disease [4].

Hematological abnormalities are common in SLE, though severe cytopenias are an unusual initial manifestation [5]. Cutaneous manifestations are diverse, with the malar rash and photosensitivity being among the most recognized presentations [6]. Here, we present an unusual cutaneous manifestation of newly diagnosed SLE in a young woman who presented with bilateral earlobe purpura associated with severe thrombocytopenia and anemia. Purpuric ear lesions in the absence of trauma have a broad differential diagnosis, including thrombocytopenic purpura, antiphospholipid syndrome, ANCA-associated vasculitis, and drug-induced vasculopathy. This case illustrates the diagnostic challenges of atypical presentations and the importance of multidisciplinary assessment when severe hematological abnormalities accompany otherwise unexplained cutaneous findings.

## Case presentation

A 31-year-old woman presented to our emergency department with a two-week history of purple discoloration of her earlobes (Figure 1), which prompted her to seek medical attention. Examination revealed bilateral earlobe purpura, with lesions measuring 2-10 mm in length, without trauma; the lesions were non-tender and had mild scaling of the overlying skin. No purpura or ecchymosis was present elsewhere on the body. She was of East Asian ethnicity and had no significant past medical history, regular medications, or travel history. On further questioning, she reported fatigue over the preceding week, accompanied by epistaxis, bleeding of the gums, and intermenstrual bleeding. The initial full blood count showed a platelet count of 8 x 10⁹/L and normocytic anemia (hemoglobin 92 g/L) (Table 1). In the context of severe thrombocytopenia, the ear lesions may have represented purpura secondary to impaired platelet-mediated hemostasis, although no histological examination was performed to establish the specific etiology. The team suspected immune thrombocytopenia (ITP) and, after discussion with hematology specialists, initiated intravenous immunoglobulin (IVIg) and tranexamic acid.

**Figure 1:**
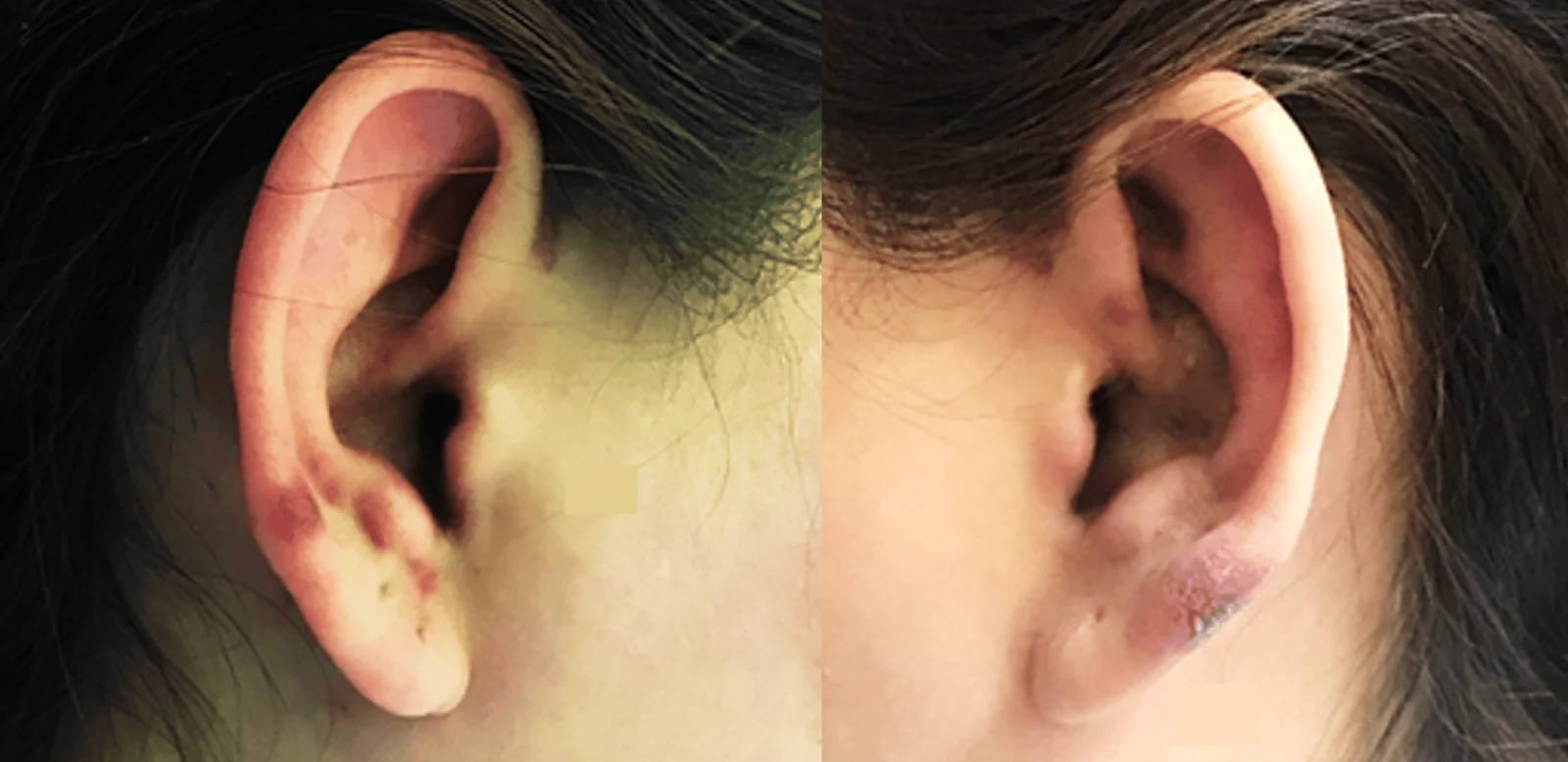
Bilateral earlobe purpura on Day 1 of admission

**Table 1:** Blood test results on Day 1 of admission APTT: activated partial thromboplastin time, CRP: C-reactive protein, INR: international normalized ratio

Blood parameters	Patient values	Reference range
White cell count (× 10⁹/L)	3.2	3.0-10.0
Red cell count (× 10¹²/L)	3.3	4.0-5.2
Hemoglobin (g/L)	92	115-155
Hematocrit (L/L)	0.28	0.33-0.45
Mean corpuscular volume (fL)	85.5	80.0-99.0
Platelet count (× 10⁹/L)	8	150-400
Neutrophils (× 10⁹/L)	1.8	2.0-7.5
Lymphocytes (× 10⁹/L)	1.1	1.2-3.7
Monocytes (× 10⁹/L)	0.2	0.2-1.0
Basophils (× 10⁹/L)	0	0.0-0.1
CRP (mg/L)	<0.6	0-4.9
Total bilirubin (µmol/L)	4	0-20
Alanine aminotransferase (U/L)	32	10-35
Aspartate aminotransferase (U/L)	45	10-35
Alkaline phosphatase (U/L)	74	30-130
Total protein (g/L)	69	60-80
Albumin (g/L)	37	35-50
Sodium (mmol/L)	140	133-146
Potassium (mmol/L)	4	3.5-5.3
Urea (mmol/L)	2.4	2.5-7.8
Creatinine (µmol/L)	51	45-84
Prothrombin time (s)	10.2	9-12
INR (ratio)	0.9	0.9-1.2
APTT (s)	35.2	29-36
Derived fibrinogen (mg/dL)	326	180-400

A peripheral blood smear confirmed thrombocytopenia without platelet clumping and showed normal red blood cell morphology. Neutrophils appeared slightly dysplastic, demonstrating hypogranulation and abnormal lobulation; a few reactive lymphocytes were seen. Viral testing was negative for active hepatitis virus infection, human immunodeficiency virus, cytomegalovirus, and Epstein-Barr virus. The hemoglobinopathy screen was also normal; therefore, the cause of the bicytopenia remained unclear.

Given increasing concern for a hematological malignancy, CT imaging of the neck, thorax, abdomen, and pelvis was performed, which demonstrated enlarged bilateral cervical, axillary, upper mediastinal, and bilateral iliac lymph nodes. These findings raised concern for a lymphoproliferative malignancy. Bone marrow aspiration and trephine biopsy were performed, along with an ultrasound-guided cervical lymph node biopsy. Rheumatology specialists were consulted and advised that vasculitis was unlikely to produce the degree of hematological abnormalities observed, specifically the thrombocytopenia. The differential diagnosis included SLE given the combination of cutaneous findings and hematological abnormalities.

The diagnostic work-up focused on identifying the underlying cause of the severe bicytopenia, rather than obtaining biopsies of the isolated ear purpura. Autoimmune serology was strongly positive, with a speckled ANA (1:5120) and positive anti-Sm and anti-dsDNA antibodies (>350 IU/mL). Complement levels were low, with C3 at 0.22 g/L and C4 at 0.03 g/L. Urinalysis showed an elevated urine albumin-creatinine ratio of 23 mg/mmol (0-2.9 mg/mmol) and an elevated urine protein-creatinine ratio of 49 mg/mmol (0-13 mg/mmol). Furthermore, bone marrow aspirate morphology and flow cytometry showed no evidence of hematological malignancy. Lymph node histology demonstrated reactive changes without evidence of malignancy. Five days after the initial hospital presentation, the acute medicine, hematology, and rheumatology teams agreed on a working diagnosis of SLE.

Hematology initiated treatment for the thrombocytopenia with IVIg; however, the patient showed little to no response. Two days into admission, prednisolone 60 mg once daily was started, and a platelet transfusion was given, but the platelet count and hemoglobin concentration remained low. By Day 5, after agreeing on the working diagnosis of SLE, methylprednisolone was initiated, with plans to start mycophenolate mofetil the following week. Rituximab was to be considered if the platelet count did not improve. Despite completing a three-day methylprednisolone pulse, the patient continued to have severe thrombocytopenia and anemia. Mycophenolate mofetil was initiated at 500 mg, with plans to increase the dose to 1,000 mg twice daily.

By Day 7, the patient's ferritin level increased rapidly, while fibrinogen declined, raising concern for hemophagocytic lymphohistiocytosis (HLH). HLH is a hyperinflammatory syndrome characterized by uncontrolled activation of the immune system leading to life-threatening multiorgan failure [7]. HLH acquired in patients with autoimmune diseases (rheumatological HLH) overlaps with macrophage activation syndrome and represents the specific phenotype of secondary HLH associated with inflammatory conditions [8]. Early HLH features are nonspecific, but a key clinical triad includes the three Fs: fever, falling blood counts, and elevated ferritin. Secondary HLH, which was suspected in this case, presents diagnostic challenges; the HScore can be used to estimate the probability of a hemophagocytic syndrome [9,10]: (1) underlying immunosuppression, (2) fever, (3) organomegaly, (4) ferritin, (5) triglycerides, (6) aspartate aminotransferase, (7) fibrinogen, (8) cytopenia, and (9) hemophagocytosis.

The patient’s ferritin level was 347 µg/L on Day 2 of admission and rose to 5,823 µg/L by Day 8. On Day 8 of admission, the patient’s Hscore was 172 points, corresponding to an estimated 54-70% probability of HLH. This was calculated using the patient's parameters on Day 8: no underlying immunosuppression, normal temperature, no organomegaly, an elevated ferritin level of 5,823 µg/L (13-150 µg/L), an elevated triglyceride level of 7.4 mmol/L (0-2.2 mmol/L), an elevated aspartate aminotransferase level of 52 U/L (10-35 U/L), normal fibrinogen of 1.7 g/L (1.5-4 g/L), bicytopenia (hemoglobin 67 g/L and platelets 22 × 10⁹/L), and no evidence of hemophagocytosis on bone marrow aspirate. Given the elevated Hscore and risk of further deterioration, the local tertiary center urgently authorized rituximab. Rituximab is a monoclonal antibody that reduces CD20+ B-cell numbers. This reduces autoantibody production in the underlying autoimmune SLE process, limiting further tissue damage and inflammation associated with HLH while improving immune-mediated hematological manifestations, including thrombocytopenia [11]. In the four days after intravenous rituximab 1,000 mg, the patient’s platelet count more than tripled (from 19 x 10⁹/L on Day 11 of admission to 68 x 10⁹/L on Day 15; Figure 2), her hemoglobin stabilized, and ferritin fell to 993 µg/L. She was discharged 16 days after admission, with a second infusion of rituximab 1,000 mg due 14 days after the first.

**Figure 2:**
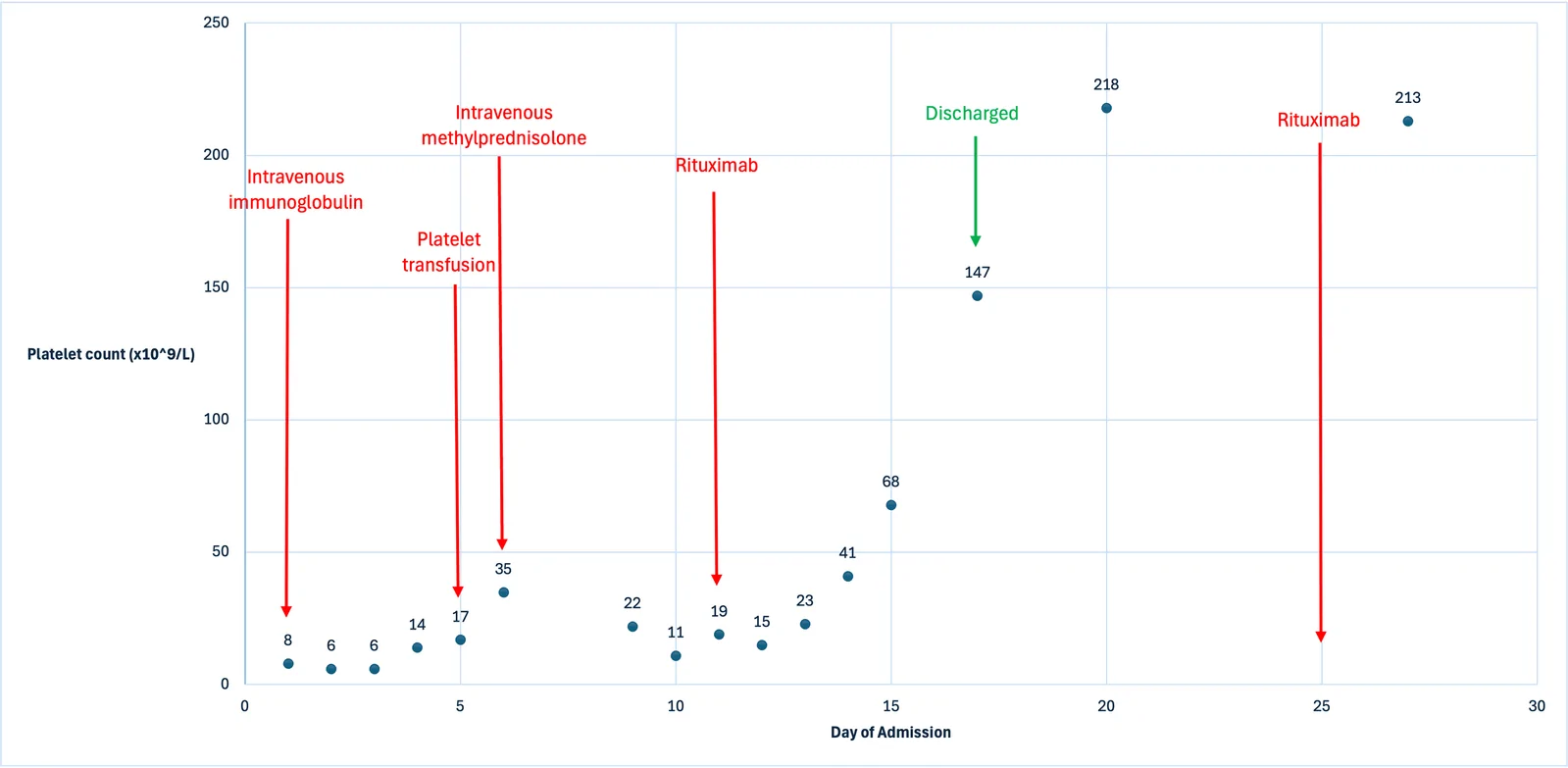
Platelet count during hospitalization and following therapeutic interventions Reference range: 150-400 x 10⁹/L

Ten months after her initial presentation, she remains on mycophenolate mofetil 1,000 mg twice daily and hydroxychloroquine 200 mg once daily; she has been weaned off oral prednisolone. The most recent blood results show stable hemoglobin of 127 g/L, and platelets have been stable at >200 x 10⁹/L for several months.

## Discussion

This case highlights the diverse clinical presentations of SLE; the patient’s presenting complaint of purple earlobes is an unusual cutaneous manifestation. Cutaneous lupus erythematosus can be classified into three forms, each with distinct clinical features. Acute cutaneous lupus erythematosus is accompanied by a malar rash in 52% of patients at diagnosis [12]. In contrast, chronic cutaneous lupus erythematosus appears as round erythematous patches, typically on the face, ears, and scalp. Finally, subacute cutaneous lupus erythematosus typically presents with a photosensitive rash that can resemble eczema or psoriasis on the neck, upper back, and extensor surfaces of the arms [13]. A limited number of reports have described persistent scaly ear rashes as a heralding cutaneous symptom of juvenile SLE [14,15]. In adult presentations, reports describe an erythematous rash of the earlobes, but only in combination with rashes on the hands and nose, more pronounced systemic symptoms [16], or neurological disturbance [17]. Thus, earlobe purpura is an unusual first feature of SLE, particularly without other skin lesions. In this case, the ear lesions were not biopsied, as the autoimmune investigations provided sufficient evidence to establish the underlying diagnosis. The purpura may have represented thrombocytopenic purpura secondary to significant thrombocytopenia, though the absence of more widespread purpura or ecchymosis made this presentation unusual. The specific etiology of the ear lesions remains unconfirmed.

Hematological abnormalities are common in SLE, including anemia, leukopenia, lymphopenia, and thrombocytopenia [18]. Studies estimate that, at SLE diagnosis, anemia is most common (63% of patients), followed by lymphopenia (40%) [5]. Thrombocytopenia in patients with SLE is associated with higher morbidity and mortality, with approximately 1% of patients demonstrating severe thrombocytopenia, defined as platelet count <20 x 10⁹/L [19]. Though other cases of SLE first presenting with significant thrombocytopenia have been reported, they are typically accompanied by more overt evidence of bleeding such as extensive ecchymosis over the trunk and limbs and rectal bleeding [20]. In this case, severe thrombocytopenia accompanied by localized earlobe purpura, rather than extensive cutaneous purpura or ecchymosis, was an unusual feature of the initial SLE presentation.

This case posed a diagnostic challenge because it lacked characteristic SLE features. The combination of atypical cutaneous findings, severe thrombocytopenia, and lymphadenopathy necessitated consideration of a broad differential diagnosis. Initial blood results raised suspicion for ITP, but imaging raised concern for a lymphoproliferative disease. The diagnostic uncertainty necessitated a multidisciplinary approach, allowing the hematological and rheumatological aspects of the presentation to be considered in parallel. Ultimately, autoimmune serology supported a diagnosis of SLE, whilst bone marrow and lymph node histology excluded an underlying hematological malignancy. Early involvement of hematology and rheumatology teams facilitated timely escalation of treatments when the platelet response was inadequate. The inadequate response to both IVIg and corticosteroids, together with concern for the development of HLH, prompted escalation to mycophenolate mofetil and rituximab. Specialist input was crucial in guiding these treatment decisions.

## Conclusions

Hematological abnormalities are common manifestations of SLE. In this case, the severity of the bicytopenia and the lack of more typical clinical features led to a broad differential diagnosis during the diagnostic work-up, including ITP and hematological malignancy. Autoimmune serology and exclusion of hematological malignancy via bone marrow and lymph node biopsy supported the diagnosis of SLE.

This highlights the importance of considering systemic autoimmune diseases in patients who present with atypical cutaneous findings and severe hematological abnormalities. Early multidisciplinary involvement can reduce diagnostic uncertainty and allow timely escalation of treatment when initial therapies fail. Recognizing atypical presentations can help clinicians identify SLE earlier, especially when the presenting features do not immediately suggest a systemic autoimmune disorder.
